# Simulating Extreme Environmental Conditions *via* Mental Imagery: The Case of Microgravity and Weight Estimation

**DOI:** 10.3389/fpsyg.2022.913162

**Published:** 2022-06-06

**Authors:** Matteo Gatti, Rocco Palumbo, Alberto Di Domenico, Nicola Mammarella

**Affiliations:** Department of Psychological Sciences, Health and Territory, University of Chieti, Chieti, Italy

**Keywords:** mental imagery, microgravity, extreme environments, weight estimation, space environment adaptation

## Abstract

Mental imagery can be used for recreating an extreme environment experience. Here we assessed whether microgravity effects over cognition, that typically occur during a space mission, may be reproduced *via* mental imagery. Participants were randomly assigned to one of two conditions in which they were guided to imagine to be (1) in outer space or (2) in a nature scenario and subsequently estimate the weight of common objects. We found that only for those who engaged in a space scenario imagery, there was a decrease in object weight estimation compared with a prior rating. This finding is the first to indicate that the effects of weightlessness on cognition can be simulated *via* an imagery-based technique and add to the ongoing debate about the importance of trying to disentangle the effect of microgravity alone on human performance. Moreover, our findings ultimately suggest that imagery can be used as a less expensive simulated scenario for studying the impact of extreme environmental conditions over astronauts’ cognition and behavior.

## Introduction

Space can be considered par excellence an extreme environment and microgravity is one of its most relevant physical stressors in space, along with cosmic radiation and altered light/dark cycles (see Kanas and Manzey (2008) for a review; Mammarella (2020a)). One of the main features of being in space is the experience of weightlessness due to the absence of gravity (although there is always some degree of gravity around us). Weightless conditions have been shown to affect human spatially oriented behaviors due the fact that gravity no longer acts as an essential vertical reference, creating a discrepancy between vestibular, visual, and sensorimotor signals (see Kanas and Manzey (2008)). Much of what we know about the impact of microgravity on astronauts’ body and mind stems from research in real microgravity conditions such as the International Space Station (ISS) or parabolic flights. In addition, simulations such as short- and long-term head-down tilt bed rest helped to unravel microgravity effects better (Mammarella, 2020b).

Although some authors wrote about the benefits of mental and motor imagery in the context of spaceflights and space missions (Bock et al., 2015; Guillot and Debarnot, 2019; Gravano et al., 2021), few, if any, considered imagination for reproducing microgravity-like effects on Earth. However, the definition of mental imagery *per sé* indicates that this can be the case. Imagery, in fact, is the ability to generate quasi-perceptual experiences in the absence of perceptual input (Kosslyn et al., 2001); the experience of sensory information without a direct external stimulus (Pearson et al., 2015). Imagery, then, allows the simulation or the re-creation of perceptual experiences across different sensory modalities (Fisher, 2006; Kosslyn et al., 2006; Pearson et al., 2008) or the ability to generate vivid images and maintaining them for goal-directed behaviors (Morris et al., 2005). As highlighted by Grabherr and Mast (2010), mental imagery is closely connected to perception and motor behavior. It aids important processes such as perceptual anticipation, problem solving and motor simulation, all of which are critical for space travel. In addition, a series of studies (e.g., Besnard et al., 2015; Bigelow and Agrawal, 2015) have also recently shown that vestibular information may play a crucial role in mental imagery, bodily self-consciousness and self-motion perception or, vice versa, that imagery can play a crucial role in vestibular cognition (e.g., Mast and Ellis, 2015). Altogether, these data support the assumption that mental imagery should be incorporated before (or during) space missions as a ground-based simulation method for studying microgravity-like effects on human performance. In fact, due to the inherent characteristics of weightlessness conditions and the intimacy between mental imagery and vestibular cognition, it can specifically simulate the activation of vestibular, visual, and sensorimotor information as in a real weightless condition. If so, the first step would be the development of a guided-imagery session of a space environment and subsequently testing whether it might affect ‘vestibular cognition’ in the “as if” manner. This may happen because when we engage in imagination, for instance, *via* guided imagery, subjective aspects (e.g., perceptual, motor, physiological and introspective) of an event and the knowledge about it, partially reactivate (Barsalou, 2008; Palmiero et al., 2019).

In order to investigate whether the perception of weightless or, to say it better, the typical effects of weightless, can be reproduced *via* mental imagery, a group of volunteers performed a weight estimation task before and after a guided-imagery session of a space scenario. If mental imagery is effective in reproducing weightlessness-like conditions, we should expect an impact on weight estimation (as one would typically expect in space, that is, objects should be perceived as less heavy) after the guided-imagery session of a space scenario. Moreover, we expected that participants of the space condition with a higher self-reported vividness (based on median values) would have provided significantly lower estimates than low-vividness subjects. The main experimental condition (space condition) was also compared with a control condition in which another group of volunteers (n = 32) performed the same task before and after a guided-imagery session of a nature scenario.

## Method

The present study was reviewed and approved by the Ethics Committee of the Department of Psychological Sciences, Health, and Territory at the University of Chieti.

### Participants and Procedure

The sample consisted of 64 participants (65.6% females; mean age 22,8 ± 3.86 SD) recruited voluntarily among undergraduates (age > 18 years) of the “D’Annunzio” University of Chieti-Pescara. Each person provided written informed consent before participating, as indicated by the ethical standards of the Declaration of Helsinki and by the IRB approval obtained from the Departmental Ethics Committee. Experimental methods were performed following approved guidelines. The experiment was administered remotely through the E-prime Go software (pstnet.com), due to the limitations imposed by the Italian government to contain the COVID-19 infection. Participants were required to sit in a quiet room, at a distance of 60cm from the PC screen. After entering their personal information, the experimental procedure began and participants were randomized equally (*n* = 32 in each condition) into one of the two conditions: space scenario or nature scenario. Students received extra credits for their participation.

A power analysis and sample size from power calculations using the program G*Power 3.1 with α = 0.05, power = 95%, indicated that we require a total of *N* = 6 (large sized effect; *f* = 0.4), *N* = 10 (medium sized effect; *f* = 0.25), or *N* = 50 (small sized effect; *f* = 0.1), participants in total to achieve 95% power when employing the traditional 0.05 criterion of statistical significance.

### Stimulus Selection and Imagery Session

In line with the guidelines for developing an effective imagery session (e.g., Lang, 1979; Holmes and Mathews, 2010; Ji et al., 2016), our mental imagery phase included a video and a guided-imagery session. In particular, before presenting the auditory imaginative script, participants were shown a video. In fact, in line with the literature on sport performance and imagery (Smith and Holmes, 2004; Wright and Smith, 2009), combining video and imagery sessions improves performance to a greater extent than imagery alone. The rationale being that providing visual stimuli allows participants to focus more on kinesthetic aspects of movement (Holmes and Calmels, 2008).

Forty object pictures were retrieved from *The Bank of Standardized Stimuli* (BOSS, Brodeur et al., 2010); additional 10 pictures were selected *via* Google Images web search. Images were adapted for size (35 × 48) and brightness and presented in the center of the screen. A group of 147 participants previously rated them in terms of weight. From those fifty stimuli, only forty were chosen as targets for the experimental task and four items were used as filler stimuli at the start and end of the task in order to reduce primacy and recency effects. Six stimuli were excluded because of the extreme variability in the estimates provided. Two different video tracks were created by editing existing videos from the *YouTube* platform. Each one lasted 2 min and 30 s. In the space video, there was an astronaut floating in the outer space and during a parabolic flight. In the nature video, there was a man walking, jumping, and crossing a naturalistic landscape with a backpack on his shoulder. Videos had no sound. Participants were asked to silently watch the video trying to internally reproduce the physical sensations that people were experiencing.

A female researcher recorded two main tracks for the guided-imagery session (one for the space scenario and one for the nature scenario). We chose standard audio scripts to reduce the effect of other auditory variables on speech recording (e.g., mood, emphasis, and prosody of the speaker, as well as quality and duration of the session, etc.). Each track lasted about 100 s. and was made of 14 sentences with an average of 83.5 words. Each sentence was followed by a pause of 5 s aimed at fostering imaginative listening. Moreover, the audio-track ended with encouraging participants to feel and enjoy their sensations (free imaginative phase). Background sounds were deleted and pauses were added *via* the free *Audacity* software. A final sentence signaled the end of the guided-imagery, prompting participants to open their eyes and continue with the experiment. An example of the guided-imagery track for the space scenario is given below:

*“Now imagine you are in outer space. If you prefer, you can close your eyes. Look around. Feel the absence of weight. Look at floating objects. If there are other people, try navigating toward them. Imagine everything you see floating. Listen carefully to all sounds. Smell the odors. Look at the lights and appreciate the dark. Look at everything that comes your way. Feel the absence of weight. Feel how you move. Take some time to enjoy this feeling*.”

The experiment was delivered *via E-prime go* software. After collecting demographic information, participants performed the weight estimation task (t1), which consisted of evaluating the weight of the selected everyday objects’ on a 0 to 100 grams scale (VAS scale). A bar appeared on the screen underneath the object picture and volunteers had to move a red cursor with the mouse in order to respond. Subsequently, participants watched video followed by the audio instructions for the guided-imagery session. At the end, all participants performed the weight estimation task (t2) for the same objects’ pictures seen before. In both tasks, of course, stimuli were randomized.

In order to be sure that participants were engaging in imagination, we also performed a manipulation check as we asked volunteers to rate the vividness and the involvement they were experiencing during the guided-imagery on a 9-point scale (from 1 = “not at all” to 9 = “extremely”). Vividness is typically used as a non-physiological measure of mental imagery (e.g., Holmes and Mathews, 2010; Iachini, 2011; Pearson et al., 2013).

## Results

**FIGURE 1:**
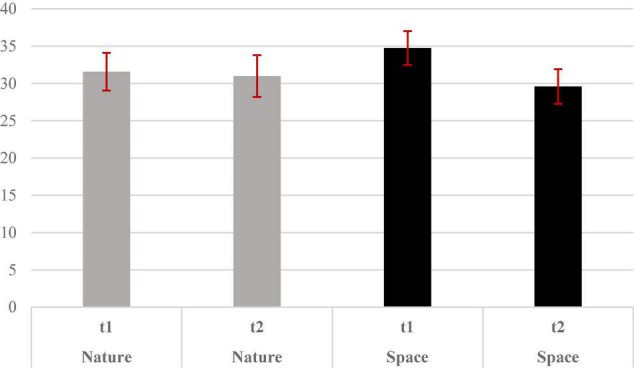
Bar chart of means and standard errors of weight estimations given by subjects in the two conditions at t1 and t2.

We conducted a 2 (type of imagery scenario: Nature vs. Space) x 2 (time of estimation: t1 vs. t2) repeated-measure ANOVA (using *Statistica version 12)* to compare the weight estimation at t1 and at t2 of participants in the space and nature scenario conditions. *Post hoc* TukeyHSD analyses were run. Results showed a main effect of time *F*_(1,62)_ = 10. 876, *p* < 0.005; ηp2 = 0.149 and a significant interaction between type of imagery scenario and time of weight estimation *F*_(1,62)_ = 6.703, *p* < 0.05; ηp2 = 0.098. Specifically, participants estimated the objects as heavier at t1 (*M* = 33.152; SE = 1.700) compared with t2 (*M* = 30.266; SE = 1.819) independently of scenario conditions. Regarding the interaction, we observed only a significant difference in weight estimation of the objects between t1 (*M* = 34.735; SE = 2.268) and t2 (*M* = 29.584; SE = 2.324; *p* < 0.001) (Figure 1 and Table 1) in the space scenario group. Thus, only participants that were engaged in a space scenario’s imagery session gave significantly lower weight estimations. No main effect of type of imagery scenario was found F_(1,62)_ = 0. 070, *p* = 0.792; ηp2 = 0.001. Specifically, the overall weight estimation in the nature condition (*M* = 31.259; SE = 2.410) was not different from the one in the space condition (*M* = 32.159; SE = 2.41).

**TABLE 1:** Means and standard errors (in parenthesis) from the weight estimation task as a function of time (t1 vs. t2) and type of imagery scenario (space vs. nature).

Imagery session	t1	t2
Space	34.735 (2.268)	29.584 (2.324)
Nature	31.570 (2.530)	30.948 (2.798)

Furthermore, a Student’s *t*-test revealed that there was no difference in vividness or involvement across the two groups and there were no significant differences in weight estimations between high and low-vividness participants (based on the median value per condition, in all cases *p* > 0.5).

## Discussion

Mental imagery is a cognitive function strictly connected to perception, visual and motor processing. It has been defined as a “weak form of perception” (Pearson, 2019) able to generate a weak form of experience, with all the neural, physiological, and psychological correlates of real perception. The primary function of imagery is to simulate reality by analyzing actions and situations’ consequences and it seems able to amplify conscious and unconscious human functions, sustain decision-making, problem-solving and even to exert control over emotions (Taylor et al., 1998; Moulton and Kosslyn, 2009; Mammarella, 2020c; Gatti et al., 2022). Due to its special features, mental imagery has been used across multiple disciplines, e.g., clinical psychology, sports and rehabilitation sciences, to cite only few. For example, in the clinical domain, Murphy et al. (2015) compared a computerized mental imagery-based training with a classical cognitive training procedure in order to modify cognitive biases in aging. In particular, an imagery-based training (see also Lang et al. (2012), Blackwell et al. (2015)) typically consisted of 12 sessions (six auditory and six visual) across four weeks. Participants in the imagery condition were asked to create a mental image of the stimuli presented. In the six auditory sessions, participants listened to brief descriptions of everyday situations and were instructed to imagine themselves actively involved and seeing the scenario through their own eyes. Each scenario was initially ambiguous, but all descriptions eventually resolved positively. In the other six sessions, participants were shown ambiguous pictures of everyday scenes paired with a few words that resolved the scene positively. Controls, instead, were asked to concentrate on the words and meanings of the descriptions, to rate the difficulty of understanding the meaning of the descriptions on a 5-point Likert-type scale, to generate a sentence by combining the picture and words, and finally rate the difficulty of generating a sentence. While both conditions improved interpretive bias modification, imagery-based training improved positive affect and vividness of positive prospective imagery (as measured by the Prospective imagery test, PIT, based on Stöber (2000)) to a greater extent. In sport sciences, instead, motor imagery has been shown to be effective in enhancing strength performance (Lebon et al., 2010; Wakefield and Smith, 2011). In this regard, the use of the PETTLEP protocol (Holmes and Collins, 2001) is very common. Specifically, PETTLEP is the acronym for Physical, Environment, Task, Timing, Learning, Emotion, Perspective, that is, the functional equivalence that imagery needs to have with the real movements to be effective. Smith et al. (2019) made a single-case design study to investigate whether combining motor imagery and action observation improves biceps strength performance in four subjects with a mean age of 24 years (SD = 3.54). Participants were assessed at baseline with the Movement Imagery Questionnaire 3 (MIQ-3, Williams et al., 2012), in which they had to reproduce visual and kinesthetic imagery of four movements (knee lift, jump, arm movement, and toe touch) and then rate their own ability on a 7-point Likert-type scale. This was followed by a phase of PETTLEP-based imaginative practice of bicep curl machine, with and without action observation, in a counterbalanced way, for a total of 8 weeks of intervention (4 weeks per intervention, with trice a week imagery session). Participants, then, completed a weekly bicep curl one repetition maximum (1 R.M.) in order to measure their performance. Combining action observation with PETTLEP imagery protocol improved performance but not significantly more than the PETTLEP alone, supporting the robustness of this motor imagery protocol per sé. In this regard, also Bock et al. (2015) highlighted the role of mental practice of some basic movements in space (or weightlessness conditions) as a relevant countermeasure technique for reducing microgravity effects.

In line with the above-mentioned studies, our goal was to understand whether an imagery-based training could also be a viable road for the study of microgravity-like effects over cognition. Of course, we are in a preliminary phase of our research and this can be considered as an exploratory study. However, the present work is the first to simulate a space mission scenario *via* imagery and to obtain significant effects on cognitive processing. Indeed, imagery is a cost-effective and non-invasive method (Guillot and Debarnot, 2019): its intrinsic simulative feature would make it an interesting device for enhancing astronauts’ performance and promoting their adaptation to extreme environmental conditions. Our hypothesis was that observing astronauts floating and inviting to imagine a weightlessness condition would lead to a reduction in weight estimation compared with imagining a nature scenario. Astronauts typically estimate the objects as less heavy in real microgravity conditions (e.g., parabolic flights, see Ross and Reschke (1982)). Here, we wanted to verify if the imagery session inducing feelings of lightness and weightlessness could affect the cognitive estimation of the weight of objects in the same manner. The rationale behind our research is rooted in the embodied cognition theories (Barsalou et al., 2003) and is oriented in investigating if a guided-imagery session of a space scenario reactivates sensorimotor aspects of weightlessness, so that participants should lower their weight estimation scores compared with controls. Our findings seem to point to this direction and suggest that imagery of a weightlessness-like condition can be effective in reactivating knowledge about space missions.

It is also important to address several shortcomings and prospects of the present study. Unfortunately, in the context of this study, it was not feasible to include real objects and compare the effectiveness of our procedure with them. Although we used a series of object pictures previously rated in terms of weight, the findings of the present research should be interpreted with caution. New research in our lab is trying to replicate findings of the current study with a design that includes real objects. Furthermore, the distinction between mass and weight of objects was not specified to participants. Weight is, in fact, the amount of gravity acting on an object mass; mass, instead, is the amount of matter that does not change with gravity. Ross and Reschke (1982), for instance, first studied mass estimation and discrimination in real microgravity conditions by asking participants to shake an object in order to generate a sensation of heaviness. An interesting avenue could be replicating these data with our guided-imagery condition. Thus, future studies should examine the effectiveness of our imaginary simulation with real objects to gain a better understanding of changes in behavioral performance. Virtual reality too may be used in this regard and provide an interesting possibility for implementing vestibular cognitive tasks. This method will additionally promote the study of integration between visual, auditory, and tactile input during simulation and increase research interest in this domain. Finally, even if the guided-imagery session seems to have an effect only in the space scenario and not in the nature scenario, our results should be interpreted with caution due to the trend of the means in the two conditions at t1. Although the weight estimation in the space scenario at t1 does not differ from the weight estimation in the nature scenario at t1, the trend of the means might suggest an apparent difference between the two tasks at baseline (before the imagery session). Therefore, further studies are needed to disentangle the effects of imagery session and potential differences due to the type of task that participants are performing. To conclude, our data first show that microgravity-like effects, that stem from an extreme environment such as space, can be reproduced *via* mental imagery and affect cognitive estimations of weight of everyday objects. Other studies adopting this methodology can help deepening our knowledge about the impact of different variables on human performance during a spaceflight. This finding, finally, can be crucial for the future of space exploration, for example, in order to understand the extent to which microgravity alone influences vestibular cognition-based tasks and adds to the development of countermeasures for long-term duration explorations (e.g., Mammarella and Gatti, 2020). Given that the number of space travelers is going to raise in next few years, we ultimately hope that our date will stimulate new research in this direction.

## Data Availability Statement

The raw data supporting the conclusions of this article will be made available by the authors, without undue reservation.

## Ethics Statement

The studies involving human participants were reviewed and approved by Departmental Ethics Committee. The patients/participants provided their written informed consent to participate in this study.

## Author Contributions

NM designed the work. MG contributed to the participants’ selection and testing. NM and MG prepared the original draft of the manuscript. RP and AD revised it critically. All authors read and approved the final version of the manuscript.

## Conflict of Interest

The authors declare that the research was conducted in the absence of any commercial or financial relationships that could be construed as a potential conflict of interest.

## Publisher’s Note

All claims expressed in this article are solely those of the authors and do not necessarily represent those of their affiliated organizations, or those of the publisher, the editors and the reviewers. Any product that may be evaluated in this article, or claim that may be made by its manufacturer, is not guaranteed or endorsed by the publisher.
